# Can vildagliptin protect against radiation-induced premature ovarian failure? Insights into the AMPK and AKT signaling pathways

**DOI:** 10.1186/s40360-025-00903-5

**Published:** 2025-04-12

**Authors:** Nada A. Mahgoub, Doaa A. El-Sherbiny, Ebtehal El-Demerdash

**Affiliations:** 1 Department of Pharmacology & Toxicology, Faculty of Pharmacy, Ain Shams University, Abbasia, Cairo Egypt; 2 Preclinical & Translational Research Center, Faculty of Pharmacy, Ain Shams University, Abbasia, Cairo Egypt

**Keywords:** Ionizing radiation, Ovaries, Vildagliptin, AKT, AMPK, CREB

## Abstract

**Background:**

Among the detrimental side effects caused by radiotherapy in young females is the ovarian damage, eventually causing premature ovarian failure (POF). While many signaling pathways contribute to the pathogenesis of POF, to date no sufficient data exist on the AMPK and AKT signaling pathways in irradiated ovaries. Both AMPK and AKT play crucial roles in the process of folliculogenesis. Vildagliptin (vilda) is a dipeptidyl peptidase-4 inhibitor with modulatory effect on both AMPK and AKT. Therefore, our study aimed to investigate the biochemical changes that occur in the AMPK/AKT signaling pathway, and the effect of co-administration of vildagliptin in radiation-induced POF.

**Methods:**

Female Sprague-dawley rats were randomly divided into four groups: control, radiation, radiation + vilda, or vilda alone groups. Vilda was administered orally once/day, and on the 10th day of the experiment, radiation and radiation + vilda group rats were subjected to 3.2 Gy of whole-body gamma irradiation. Behavioral activity was assessed on the 13th day of the experiment. On day 14 of the experiment, all rats were euthanized. Serum samples were collected, and ovaries were dissected for histological and biochemical analyses.

**Results:**

Irradiation of female rats resulted in increased locomotor hyperactivity, impaired memory, and ovarian damage as evidenced by the marked histopathological deterioration. Additionally, irradiation led to a significant decrease in body weight gain, gonadosomatic index, and serum estradiol level. Further, it caused a significant increase in serum AMH, phosphorylated AMPK, phosphorylated AKT, cytoplasmic Nrf2 expression and phosphorylated CREB levels. Co-administration of vilda exhibited neuroprotective effects, preserved the ovarian histological architecture but failed to preserve the primordial follicle pool in irradiated rats.

**Conclusion:**

In conclusion, AMPK/AKT signaling pathway is upregulated in radiation-induced POF. It possibly contributes to POF pathogenesis by accelerating the activation of primordial follicles, hence leading to their premature depletion. Coadministration of vilda can protect the ovaries and temporarily preserve its endocrine function; however, it does not sustain the ovarian reproductive capacity due to the early depletion of the pool of primordial follicles. Women undergoing radiotherapy should be cautious with the use of AKT-activating drugs.

## Graphical Abstract

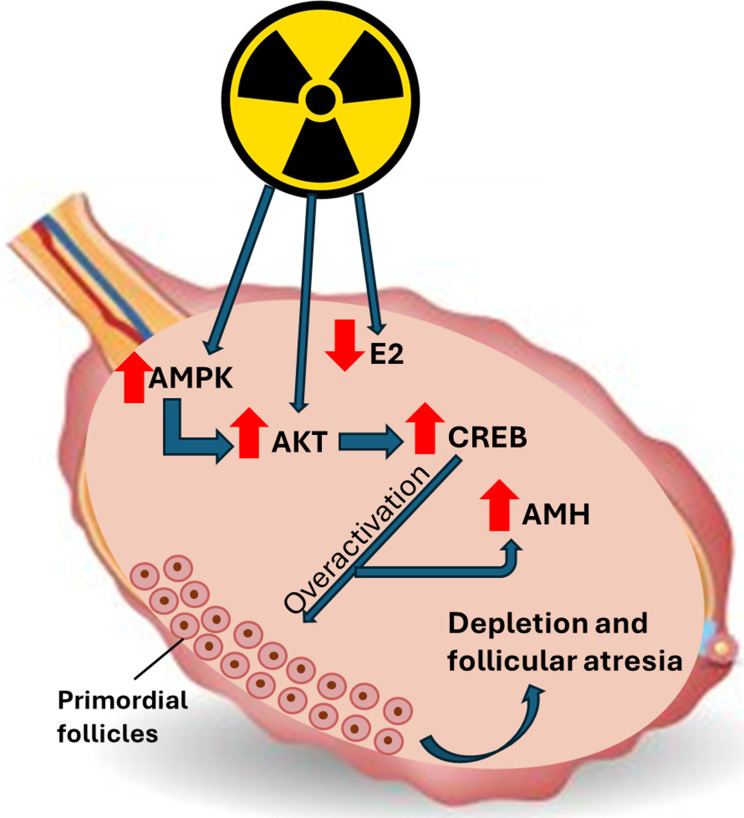

## Background

Ovaries are the cornerstone of the female reproductive system, considering their pivotal role as both a reproductive organ responsible for the release of mature oocytes, and an endocrine organ responsible for the release of estrogen (E2) hormone [1]. The regular release of mature oocytes and estrogen ensures a regular menstrual cycle for females during their reproductive life. Women undergo natural menopause at an average age of 50 years old, when they experience idiopathic amenorrhea for 12 consecutive months [2].

An impaired ovarian function or diminished ovarian reserve that occurs before the age of 40 years is referred to as premature ovarian failure (POF), or premature ovarian insufficiency [3]. POF can result in earlier than normal amenorrhea with the associated complications of menopause that are either short-term as hot flushes, night sweats, mood disorders, and low libido, or long-term complications as compromised cognition, cardiovascular, and bone health [4]. POF has a complex etiology including genetic, metabolic, autoimmune, environmental, and iatrogenic causes [5]. Radiotherapy and chemotherapy are among the main iatrogenic causes of POF, and with the increasing incidence of cancer nowadays, many females are being subjected to radiotherapy and/or chemotherapy for the diagnosis and treatment of various tumors. It is estimated that 6.6 million females are diagnosed with cancer annually, of whom 10% are younger than 40 years old, hence at risk for developing chemotherapy/radiotherapy-induced POF [6].

Unfortunately, radiotherapy has deleterious effects on different body organs. The ovaries are among the most sensitive organs to the damaging effects of radiation in females subjected to either pelvic or total body irradiation [7]. Ionizing radiation (IR) induces ovarian DNA damage either directly by incorporating lesions in the DNA double helix, or indirectly by stimulating the production of reactive oxygen species (ROS) thus causing DNA breaks [8]. Understanding the different molecular pathways occurring in the ovaries that are subjected to IR is essential to help and guide the development of novel protective and treatment options that would improve the quality of life of female cancer survivors.

One of the important, yet vague, signaling pathways in the ovaries is the AMPK/AKT pathway. AMPK (Adenosine monophosphate-activated protein kinase) is a heterotrimeric protein that is well recognized in different cells for its effect on energy production and consumption pathways. In the ovaries, AMPK is expressed by almost all ovarian cell populations to regulate primordial follicle activation [9]. AKT, also known as protein kinase B, is a serine/threonine protein kinase that is responsible for different vital cellular functions including but not limited to cell growth, survival, and proliferation [10]. AKT together with other kinases regulate primordial follicle growth and development [11]. In addition decreased fertility was encountered in AKT1-deficient female mice, highlighting its role in the mammalian ovaries [12]. Numerous studies examined the cross-talk between AMPK activation and AKT [13–15], and recent studies showed conflicting results on the role of AMPK in POF where AMPK activation provided protection against POF in one study [16], while in another study protection against POF was offered by AMPK inhibition [17]. It was also reported that under oxidative stress conditions, AMPK pathway is activated in oocytes to induce autophagy [18]. To date, no study focused on the effects of radiation exposure on AMPK/AKT pathway, and the ultimate consequence on the ovaries.

Dipeptidyl peptidase 4 (DPP-4) inhibitors are commonly prescribed oral anti-diabetic medications that includes many approved drugs such as sitagliptin, linagliptin, saxagliptin, and vildagliptin. These drugs are primarily used for the treatment of type 2 diabetes mellitus [19]. Furthermore, DPP-4 inhibitors have proven a protective potential in various models of ovarian dysfunction. For example, sitagliptin showed a protective effect against chemotherapy-induced ovarian failure [20], and polycystic ovary syndrome [21]. Also, vildagliptin (vilda), one of the modulators of both AMPK and AKT has been experimentally repurposed for the protection against various neurological, cardiac, bone, and renal disorders [22–25]. In our lab, vilda showed promising protective effects against liver fibrosis and cisplatin-induced chemo-brain through its action on both AMPK and AKT [26, 27], which made us curious to explore whether these protective effects could extend to a condition as sensitive and vital as radiation-induced POF. So, in this study we aimed to investigate the mechanistic changes of AMPK and AKT in the ovaries upon their exposure to radiation, and whether vilda could preserve the ovarian function and provide protection against radiation-induced POF.

## Materials and methods

### Animals

Female Sprague-Dawley rats (4–5 weeks old, weighing around 50–70 g) were purchased from Nile Co. for pharmaceutical and chemical industries, Egypt. Rats were housed in ventilated plastic cages with bedding, in an air-conditioned atmosphere at a temperature of (23 ± 2 °C) with alternatively 12-hour light and dark cycles at the animal facility of the Faculty of Pharmacy (Ain Shams University, Egypt). They were allowed free access to water and animal chow (containing not less than 20% protein, 5% fiber, 3.5% fat, 6.5% ash, and a vitamin mixture). The experiment was conducted in compliance with ARRIVE guidelines.

### Radiation protocol

Whole body gamma (γ)-irradiation was carried out using a Cesium-137 (^137^CS) source, (Gamma Cell-40 Biological Irradiator) at the National Center for Radiation Research and Technology (NCRRT), Cairo, Egypt.

### Experimental design

Rats were randomly divided into four groups (*n* = 15 per group): Control group, Radiation (IR) group, Radiation (IR) + vilda group, and Vilda alone group. The experiment and subsequent analyses were carried out in a blinded manner.

The rats were treated for 13 consecutive days as follows; Control and radiation groups’ rats received normal saline solution orally (the vehicle for vildagliptin), while radiation + vilda and vilda alone groups’ rats received vildagliptin orally at a dose of 10 mg/kg/day. Vildagliptin dose was selected based on previous studies [23, 27]. On the 10th day of the experiment, radiation and radiation + vilda groups’ rats were subjected to whole body γ-irradiation -one hour after the drug administration- at a single dose of 3.2 Gy, given at a dose rate of 0.48 Gy/min [28].

**Fig. 1:**
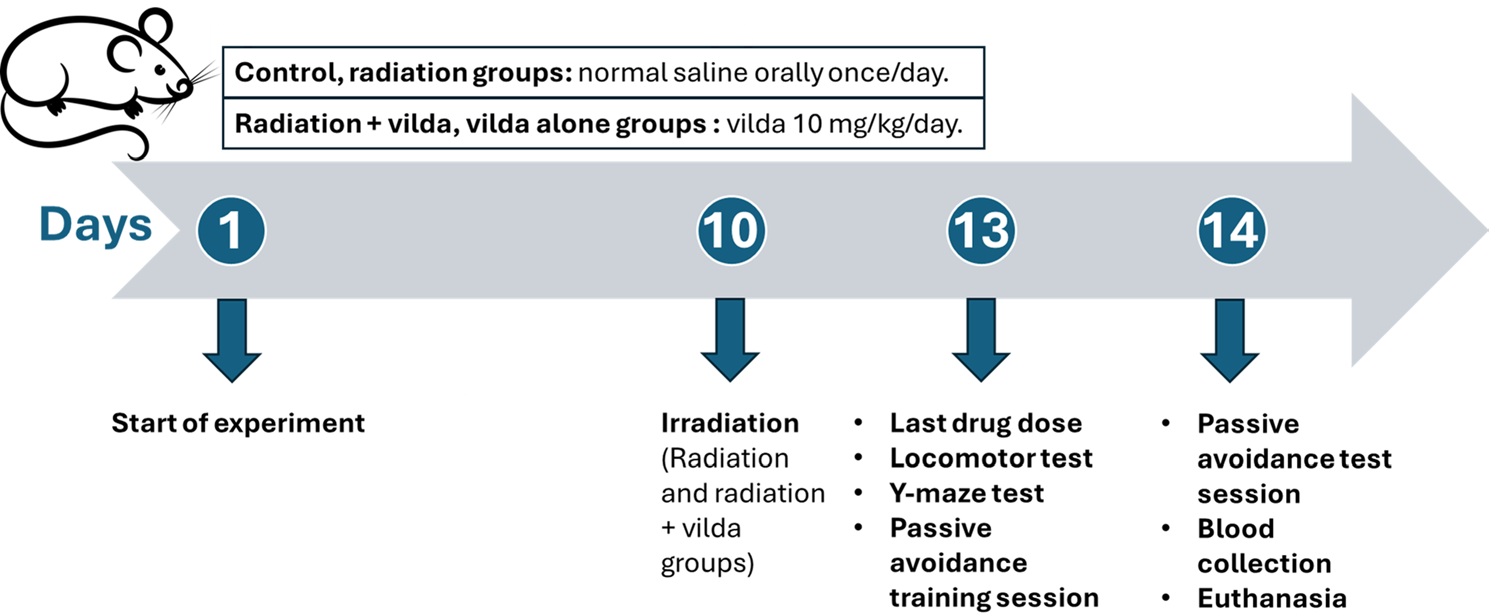
Experimental design and timeline

Twenty-four hours after the last vildagliptin dose, rats were weighed and anesthetized using ketamine hydrochloride (50 mg/kg, i.p.). Then, blood samples were collected from the retro-orbital plexus and allowed to clot. Serum was separated by centrifugation of the blood at 4000 rpm and 4 °C for 10 min and then stored at -20 °C for subsequent use in biochemical analyses. Rats were then euthanized by cervical dislocation and their ovarian tissues were dissected, washed with ice-cold saline, and weighed. Ovarian tissues (*n* = 5) from different groups were fixed in 10% buffered formalin for histopathological and immunohistochemical examination. Other ovarian tissues (*n* = 10) were homogenized in ice-cold phosphate-buffered saline (PBS, pH 7.4, molarity 50 mM/L) to prepare a 10% homogenate (w/v) which was stored at -80 °C for subsequent biochemical analyses (Fig. 1).

### Behavioral assessment

The following tests were conducted to assess the behavioral changes in the rats after being exposed to radiation.

#### Locomotor activity test

The locomotor activity test was used to assess the spontaneous locomotor activity and arousal in rats exposed to IR. An activity monitor (Opto-Varimex-Mini Model B, Columbus Instruments, Columbus, OH, USA) was used, which is equipped with 15 infrared beams (wavelength = 875 nm, diameter = 0.32 cm, spaced 2.65 cm apart, and scan rate = 160 Hz). The locomotor activity can be reliably detected based on the number of beam interruptions. Each rat was monitored for 5 min and the locomotor activity was presented as no. of counts / 5 min [29].

#### Y-maze test

The effect of radiation exposure and vilda co-administration on short-term memory in rats was assessed using the Y-maze test which employs a Y-shaped device with 3 identical opaque arms (named A, B, and C) with dimensions of 40 cm length x 8 cm width x 15 cm height. As a measure of spatial working memory, spontaneous alteration was assessed by allowing rats to freely explore the 3 arms of the maze throughout a time interval of 5 min. By nature, a rat with an intact working memory would be eager to enter a less recently visited arm. A rat had to step into the arm with its 4 paws to be considered as a valid entry.

The total number of alternations and total arm entries (TAE) were recorded, then the spontaneous alternation percentage (SAP) was calculated according to the following formula: “the total number of alternations” divided by “the total possible alternations (i.e. the total number of arm entries minus 2)” and multiplied by 100. i.e. SAP= [(number of alternations)/ (TAE − 2)] × 100 [30, 31].

#### Passive avoidance test

The passive avoidance test, also referred to as step-through latency, was used to assess the connecting memory of rats as they learn to avoid a painful stimulus, using a step-through apparatus (UGO Basile, Italy). The device consists of 2 chambers separated by a sliding door, an illuminated one and a dark one equipped with stainless steel shock inducing bars. Each rat was placed in the device twice on 2 consecutive days, once for training session and then test session. On the 13th day of our experiment and following the administration of the last vilda dose, rats were trained by placing them in the illuminated chamber then the sliding door opens allowing them to enter the dark chamber where a very brief electric shock is delivered to the rat’s paws. A rat hereby learns that it will get shocked upon entering the dark chamber. On the next day, 24 h after the training session, rats were placed in the illuminated room to assess their memory retrieval ability. The time it takes a rat to enter the dark room is the step-through latency (STL). A rat would be considered having a good memory if it didn’t enter the dark chamber, the cut-off time was 5 min [32].

### Assessment of body weight change and gonadosomatic index (GSI)

The change in the body weight of the rats was calculated according to the formula: Change in body weight = Final weight – initial weight.

GSI, can also be referred to by relative ovarian weight, is calculated as the gonads’ weight (ovaries in our case) relative to the total body weight, according to the formula: GSI = (Ovarian weight/Body weight) * 100 [33].

### Histopathological examination

Ovarian tissue specimens collected from rats were fixed in 10% neutral buffered formalin. The fixed specimens of the ovaries were then trimmed, washed, and dehydrated in ascending grades of alcohol (methyl, ethyl, and absolute ethyl). They were then cleared in xylene, embedded in paraffin, sectioned at 4–6 μm thickness, and stained by hematoxylin and eosin (H & E) stain following Bancroft’s techniques [34]. The stained slides were examined by digital light microscope (Olympus xc30, Tokyo, Japan).

### Morphometric analysis of follicles population

In all H & E-stained ovarian samples, the fifth cut was used for counting ovarian follicles which were then classified as either primordial (contained an oocyte surrounded by flattened pre-granulosa cells), preantral (contained an oocyte with more than one layer and less than five layers of granulosa cells without an antral space), antral (contained an oocyte with more than five layers of granulosa cells and/or antral space), or atretic (contained a degenerating oocyte or pyknotic granulosa cells) [35].

### Assessment of the serum levels of estradiol (E2) and anti-Müllerian hormone (AMH)

In order to explore the effects of vilda on hormone levels in radiation-induced POF, serum levels of E2 and AMH were assessed using commercial ELISA kits; E2 (Catalog no.: 10009, Chemux Bioscience, Inc., USA), and AMH (Catalog no.: SL0504Ra, SunLong Biotech Co., Zhejiang, China).

### Immunohistochemical detection of nuclear factor erythroid 2-related factor 2 (Nrf2)

Paraffin-embedded tissue sections of 3 μm thickness were rehydrated first in xylene and then in graded ethanol solutions. The slides were then blocked with 5% bovine serum albumin in tris-buffered saline for 2 h. The sections were then immunostained with Nrf2 primary antibody: rabbit polyclonal anti-rat Nrf2 antibody (ABclonal, Cat No. A0674) at a concentration of 1 µg/ml containing 5% bovine serum albumin in tris-buffered saline and incubated overnight at 4 °C. After washing the slides with tris-buffered saline, the sections were incubated with goat anti-rabbit secondary antibody. Sections were then washed with tris-buffered saline and incubated for 5–10 min. in a solution of 0.02% diaminobenzidine containing 0.01% H_2_O_2_. Counter staining was performed using hematoxylin, and the slides were examined using light microscope. Quantitative image analysis for the immunostaining was done using Image J image analysis software (NIH, USA) and was expressed as optical density (O.D.).

### Assessment of the signaling pathway pAMPK/pAKT/pCREB

The effects of vilda on the AMPK/AKT/CREB pathway in radiation-induced POF were assessed by measuring the levels of the phosphorylated forms of AMPK, AKT, and CREB respectively in the ovarian tissue homogenate using commercial ELISA kits. pAMPK (Catalog no.: CSB E14394m, Cusabio, Houston, USA), pAKT (Catalog no.: SG-20457, SinoGeneClon Biotech Co., China), and pCREB (Catalog no.: EK770311, AFG-Bioscience, USA).

### Data and statistical analysis

Data are presented as mean ± S.D. Multiple comparisons were performed using one-way ANOVA followed by Tukey-Kramer as a post-hoc test. The multiple comparisons in passive avoidance test were performed using Kruskal-Wallis test followed by Dunn’s post-hoc test. The 0.05 level of probability was used as the criterion for significance. All statistical analyses were performed using GraphPad Instat version 3.06 software package. Graphs were sketched using GraphPad Prism (ISI^®^ software, USA) version 5 software.

## Results

### Effect of vilda cotreatment on radiation-induced behavioral changes

#### Locomotor activity test

**Fig. 2:**
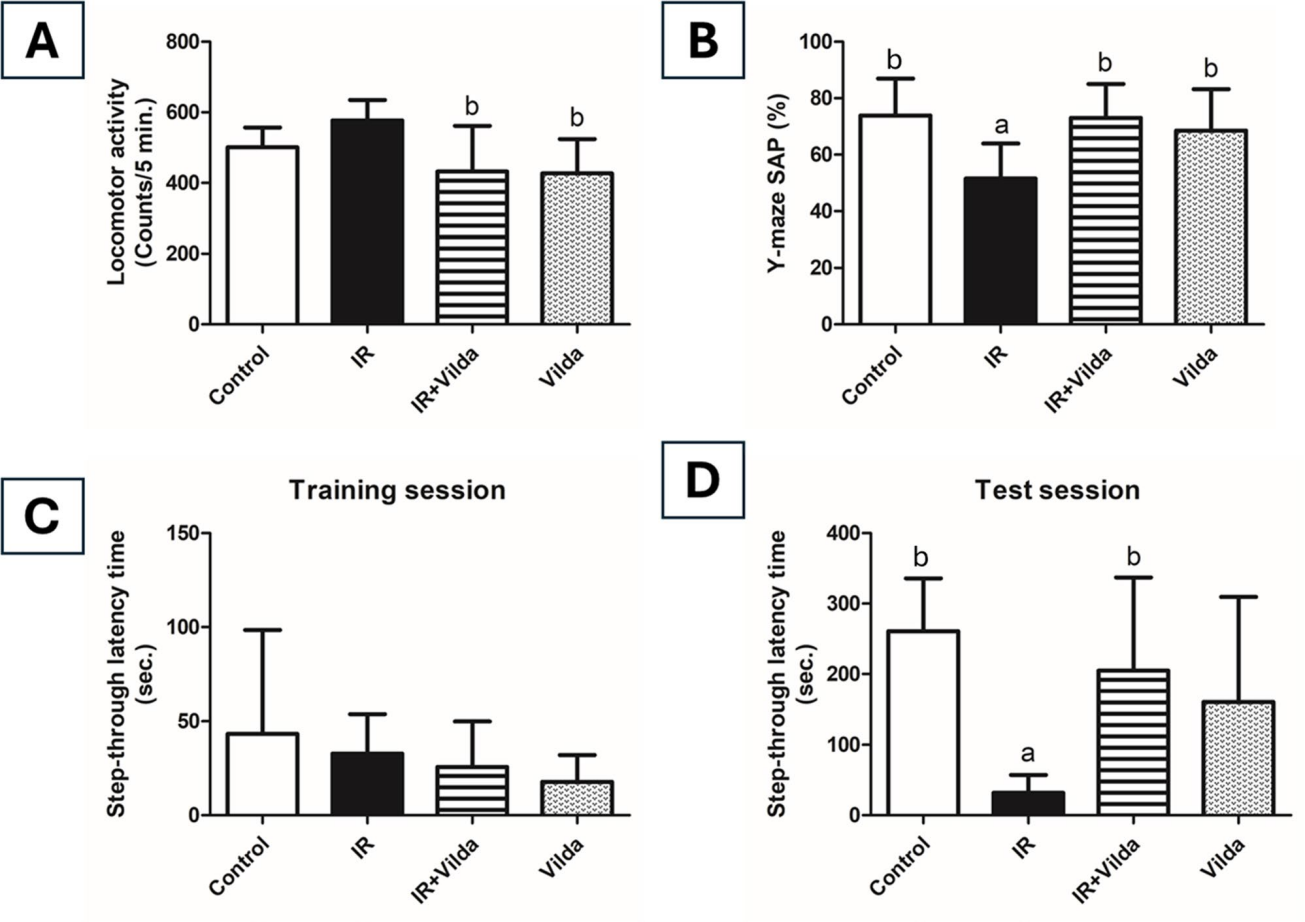
Effect of vildagliptin co-administration on radiation-induced behavioral changes. (**A**) Locomotor activity. (**B**) SAP% in Y-maze test. Data are presented as mean ± S.D (*n* = 8), a; significantly different from control group at *p* < 0.05, b; significantly different from radiation group at *p* < 0.05 using one-way ANOVA followed by Tukey-Kramer as a post-hoc test. (**C**) Passive avoidance test; Step-through latency time in training session. (**D**) Passive avoidance test; Step-through latency time in test session. Passive avoidance test data are presented as mean ± S.D (*n* = 8), a; significantly different from control group at *p* < 0.05, b; significantly different from radiation group at *p* < 0.05 using Kruskal-Wallis test followed by Dunn’s post-hoc test

Exposure of rats to IR increased their spontaneous locomotor activity by 15.2% as compared to the control group, however this increase was statistically insignificant. Co-administration of vilda was able to restore the normal locomotor activity as of control rats. Vilda-alone treated rats showed no significant difference from the control group (Fig. 2A).

#### Y-maze test

Short-term memory was obviously impaired in irradiated rats as evidenced by the significant decrease in the percentage of spontaneous alteration by 30% as compared to control rats. Vilda co-administration was able to improve short-term memory by significantly increasing SAP% by 41.57% as compared to irradiated rats. Treatment with vilda alone showed no significant change from the control group (Fig. 2B).

#### Passive avoidance test

In the training session, there was no significant difference in step-through latency time between rats from all groups (Fig. 2C). In the test session, rats exposed to IR suffered from impaired memory acquisition and retrieval abilities as evidenced by the significant decrease in latency time by 87.8% as compared to control rats. Memory enhancement was observed upon co-administration of vilda where STL time was significantly increased by 6.44 folds as compared to irradiated rats. Treatment with vilda alone didn’t show any significant change from the control group (Fig. 2D).

### Effect of vilda cotreatment on body weight and GSI

Table 1 shows that exposure of female rats to γ-radiation caused a significant decrease in both the change in body weight and the relative ovarian weight by 42% and 26%, respectively as compared to control rats. Co-administration of vilda was able to significantly increase the change in body weight as compared to radiation group, however it still showed a significant difference from the control group. In addition, the relative ovarian weight showed a significant decrease in radiation, treatment, and vilda alone groups as compared to the control group.

**Table 1:** Effect of vildagliptin co-administration on change in body weight and GSI in radiation-induced POF

Group	Control	Radiation	Radiation + Vilda	Vilda alone
Change in body weight (gm)	39.5 ± 7.9	21.3 ± 4.4^a^	28.7 ± 6.9^a, b^	40.4 ± 9.1^b^
GSI (%)	0.078 ± 0.015	0.063 ± 0.009^a^	0.057 ± 0.014^a^	0.056 ± 0.017^a^

a. Female rats were subjected to a single dose of 3.2 Gy whole-body γ-radiation, and/or vildagliptin (10 mg/kg/day, orally) for 13 consecutive days

b. Data are presented as mean ± S.D (*n* = 15)

c. a; significantly different from the control group at *p* < 0 0.05, b; significantly different from radiation group at *p* < 0 0.05, using one-way ANOVA followed by Tukey-Kramer as a post-hoc test

### Effect of vilda cotreatment on ovarian histopathology

**Fig. 3:**
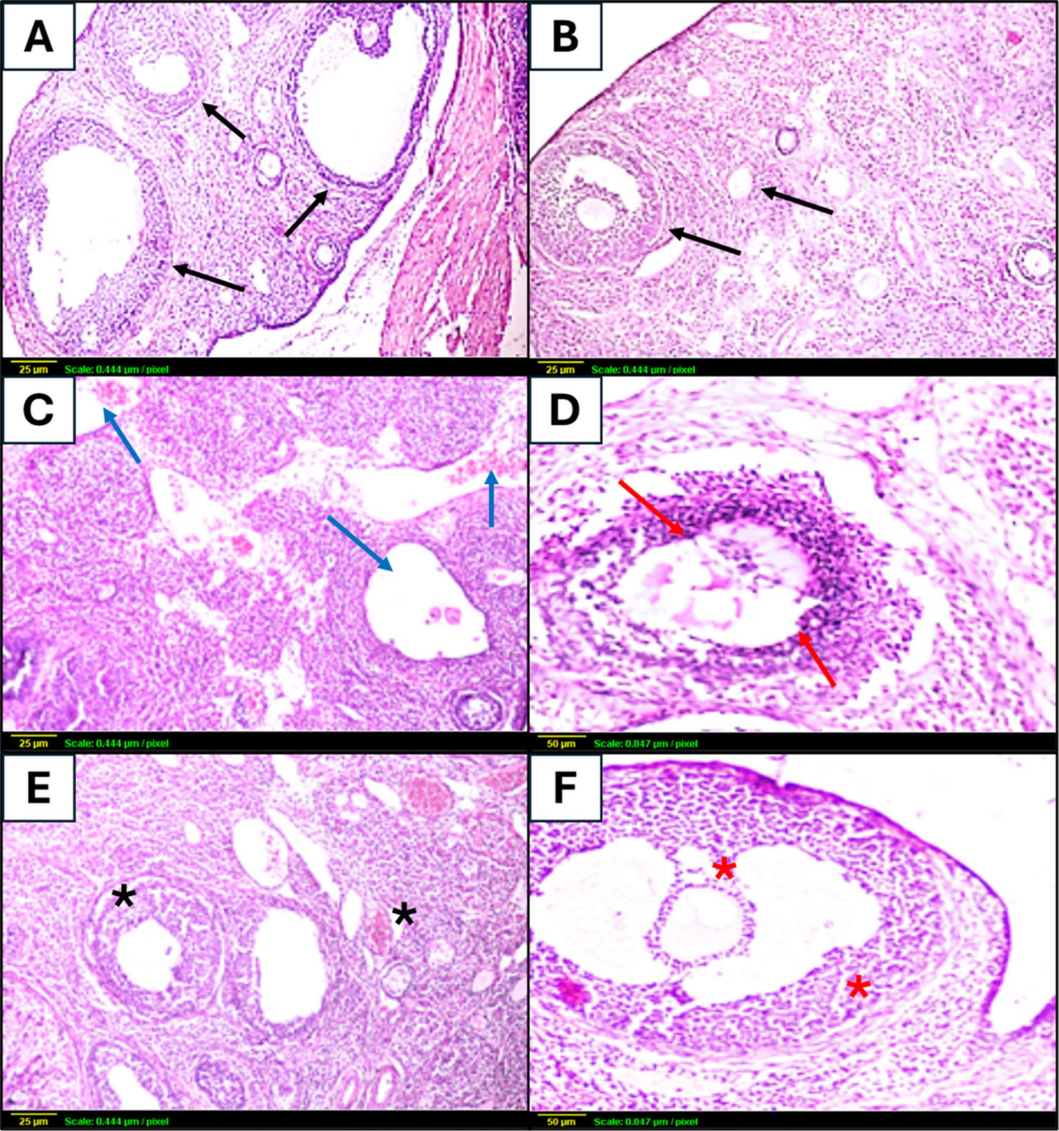
Effect of vildagliptin co-administration on radiation-induced histological alterations of the ovarian tissue using H & E staining. (**A**) Control ovaries showing normal histological architecture, ovarian follicles in various stages of development (black arrows) (x 200). (**B**) Ovaries from vilda-alone treated rats showing normal histological architecture, ovarian follicles in various stages of development (black arrows) (x 200). (**C**) Irradiated ovaries showing hyperplasia of ovarian connective tissue, marked dilatation of ovarian blood vessels, and leukocytic infiltration (blue arrows) (x 200). (**D**) Irradiated ovaries showing pyknosis and degradation of granulosa cell layers of graafian follicles and disorganization of internal thecal layer (red arrows) (x 400). (**E**) Ovaries from IR + vilda rats where mild oedema of interstitial tissue and mild congestion of ovarian blood vessels were observed (black asterisk) (x 200). (**F**) Ovaries from IR + vilda showing a mature graafian follicle with a centrally-located oocyte and normal organization of granulosa cells (red asterisk) (x 400)

Ovarian tissue sections from control and vilda alone rats showed normal histological architecture without considerable pathological alterations. The ovarian cortex contained follicles at various developmental stages. Normal ovarian follicles including primary and tertiary follicles were seen, in addition to graafian follicles with centrally located oocyte. The cuboidal follicular cells around the oocyte were arranged in a regular manner (Fig. 3A and B).

Exposure to γ-radiation induced marked pathological deterioration of the ovaries. Atrophy in both cortical ovarian follicles and medulla layers was observed together with a marked decrease in the number of ovarian follicles. Large corpora luteal cysts and many degenerated primary follicles were seen. Pyknosis and degradation of granulosa cell layers of graafian follicles and disorganization of internal thecal layer were observed. Ovarian connective tissue showed profound hyperplasia, along with marked dilatation of ovarian blood vessels with leukocytic infiltration -mostly lymphocytes and macrophages- (Fig. 3C and D).

Vilda co-administration in irradiated rats offered a marked protection of ovarian parenchyma. Multiple follicles in different developmental stages and well organized graafian follicle with oocyte were noticed in the cortical layer. Mild edema of interstitial tissue and mild congestion of ovarian blood vessels with little number of leucocytic cell infiltration were seen. Mature graafian follicle with centrally-located oocyte was also seen (Fig. 3E and F).

### Effect of vilda cotreatment on ovarian follicles count

**Fig. 4:**
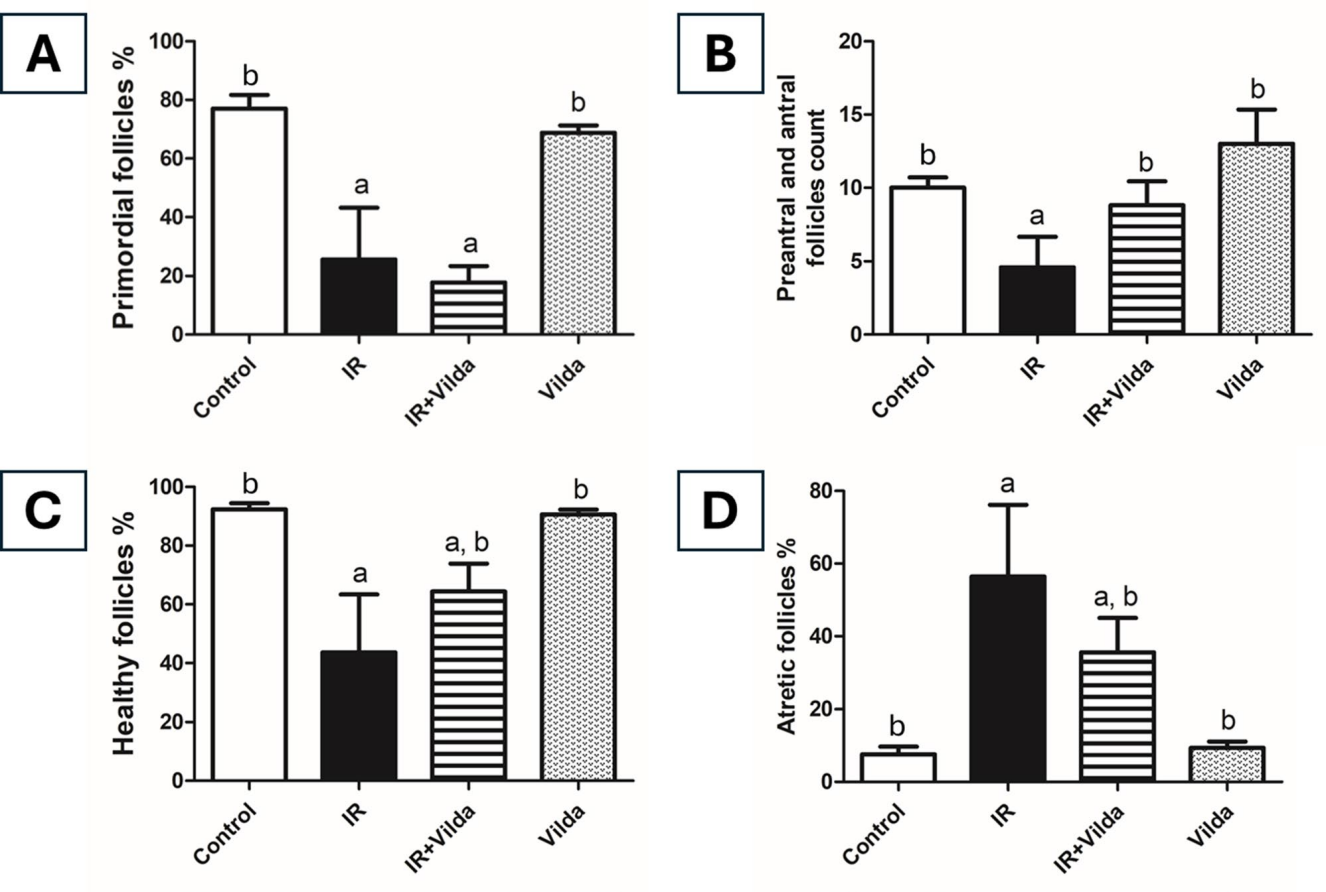
Morphometric analysis of ovarian follicles population. (**A**) Primordial follicles percentage. (**B**) Preantral and antral follicles count. (**C**) Healthy follicles percentage. (**D**) Atretic follicles percentage. Data are presented as mean ± S.D (*n* = 5), a; significantly different from control group at *p* < 0.05, b; significantly different from radiation group at *p* < 0.05 using one-way ANOVA followed by Tukey-Kramer as a post-hoc test

Morphometric analysis of follicular population revealed a marked and significant decrease in primordial follicles pool and healthy follicles percentage in γ-radiated ovaries by 66.75% and 52.84% respectively as compared to control ones, as well as a significant increase in the percentage of atretic follicles by 7.42 folds. Co-administration of vilda showed a further decrease in the primordial follicles pool by 76.88% and 30.47% as compared to the control and radiation groups, respectively. The observed decrease upon vilda co-administration varied significantly from the control group but not from the radiation group. However, it showed a significant increase in the percentage of healthy follicles as well as a significant decrease in the percentage of atretic follicles, as compared to both control and radiation groups. Vilda alone didn’t show any significant changes from the control group in the percentages of both healthy and atretic follicles (Fig. 4).

### Effect of vilda cotreatment on hormone levels

**Fig. 5:**
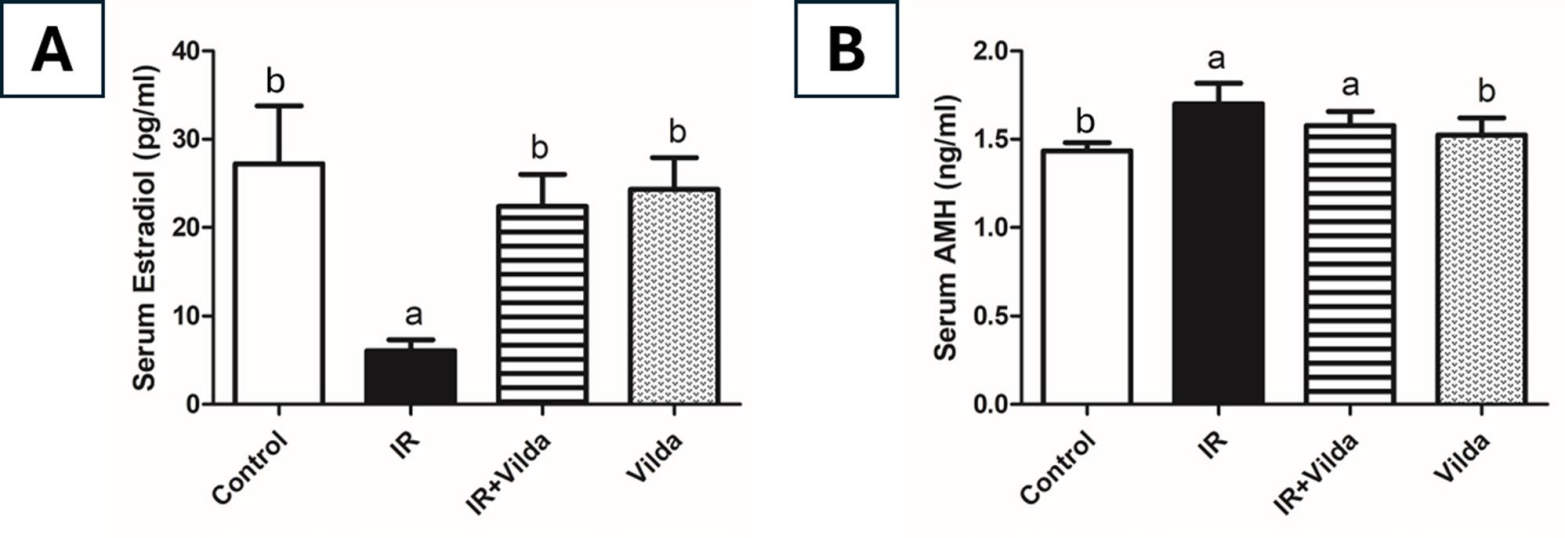
Effect of vildagliptin co-administration on radiation-induced hormonal changes. (**A**) Serum estradiol level. (**B**) Serum AMH level. Data are presented as mean ± S.D (*n* = 5), a; significantly different from control group at *p* < 0.05, b; significantly different from radiation group at *p* < 0.05 using one-way ANOVA followed by Tukey-Kramer as a post-hoc test

Gamma radiation exposure markedly affected serum hormone levels where it significantly reduced serum E2 level by 78%, while significantly elevated serum AMH level by 19% as compared to the control group. Co-treatment with vilda was able to restore the normal control level of E2, however it caused a significant increase in AMH level as compared to control group. Vilda alone administration didn’t show any significant changes from the control group (Fig. 5).

### Effect of vilda cotreatment in Nrf2 expression

**Fig. 6:**
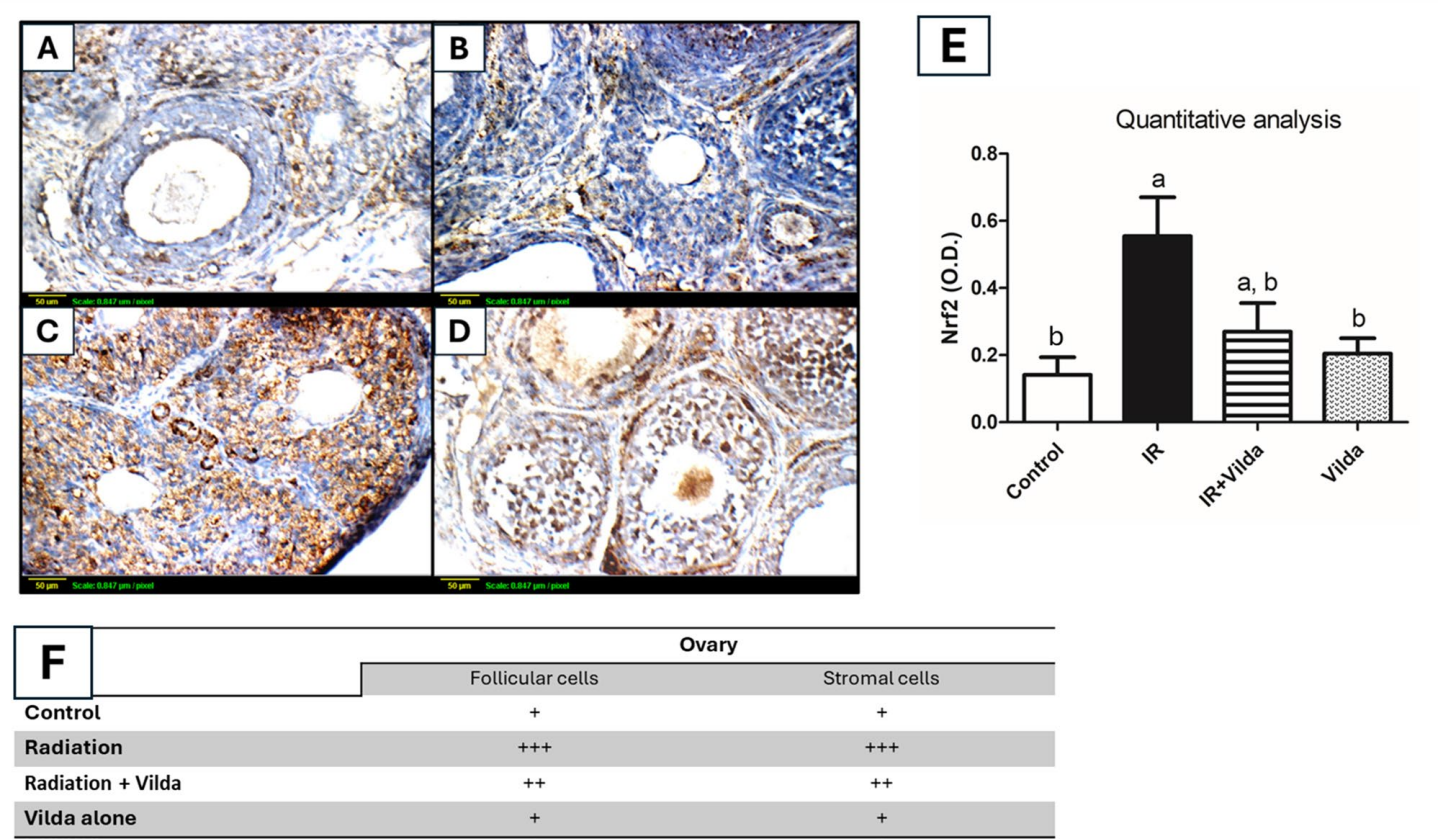
Immunohistochemical detection of Nrf2 expression (x 400). (**A**, **B**) Control and vilda alone groups showed minimal immunostaining, respectively. (**C**) Ovaries from irradiated rats showed extensive brown immunostaining (increased cytoplasmic Nrf2). (**D**) Vilda co-administration resulted in a marked reduction in cytoplasmic Nrf2. (**E**) Quantitative image analysis for Nrf2 immunostaining was expressed as optical density (O.D.). (**F**) Scoring of the immunostaining considering both the staining intensity and the percentage of positive cells

As shown in Fig. (6), Nrf2 cytoplasmic staining was considered as positive, and the interpretation of the results considered both the staining intensity and the percentage of positive cells in both follicular and stromal cells. The reactivity was classified as negative (0), weak (+), moderate (++), or marked (+++). Ovaries from control and vilda alone groups showed weak cytoplasmic reactivity for Nrf2 in follicular and stromal cells. However, ovaries from irradiated rats showed marked cytoplasmic reactivity for Nrf2 in both follicular and stromal cells. Quantitative analysis -expressed as O.D.- revealed a significant increase in cytoplasmic Nrf2 in irradiated rats by 3.9 folds, as compared to control rats. Vilda co-administration was able to significantly decrease cytoplasmic Nrf2 by 51.34%, as compared to radiation group. Treatment with vilda alone didn’t show any significant change from the control group.

### Effect of vilda cotreatment on AMPK and AKT signaling pathways

**Fig. 7:**
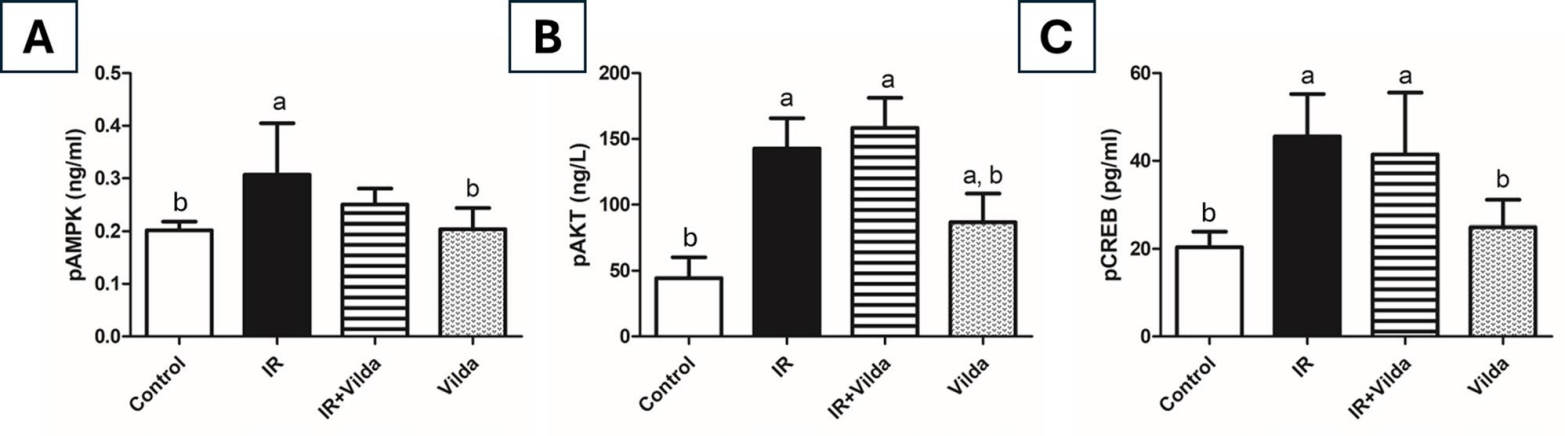
Effect of vilda co-administration on radiation-induced changes in AMPK/AKT/CREB signaling pathway. (**A**) Ovarian tissue level of phosphorylated AMPK. (**B**) Ovarian tissue level of phosphorylated AKT. (**C**) Ovarian tissue level of phosphorylated CREB. Data are presented as mean ± S.D., a; significantly different from control group at *p* < 0.05 using one-way ANOVA, b; significantly different from radiation group at *p* < 0.05 using one-way ANOVA followed by Tukey-Kramer as a post-hoc test

AMPK signaling was noticeably affected by exposure to γ-radiation where this ionizing radiation was able to switch on the AMPK pathway as shown by the significantly elevated level of pAMPK by 1.5 folds as compared to control rats. That rise in pAMPK was also accompanied by a significant elevation of pAKT level by 3.2 folds, which eventually led to the significant elevation of pCREB level by 2.2 folds as compared to the control group. Co-administration of vilda resulted in a considerable decrease in pAMPK level that differed insignificantly from both control and radiation groups. However, it led to a significant 3.6-fold increase in pAKT level together with a 2-fold increase in pCREB level as compared to the control group. Vilda alone administration showed no significant differences from the control group except in pAKT level (Fig. 7).

## Discussion

Nowadays exposure to radiation is inevitable, being used in the diagnosis of a wide range of ailments, and the treatment of different tumors. Hence, exploring and understanding the biochemical changes that happen in the cells upon radiation exposure can guide us towards developing successful strategies for protection against possible radiation hazards [36].

Radiation-induced POF is a major health concern since it affects the reproductive life of young female cancer survivors. Its pathogenesis involves many signaling pathways, among the mysterious ones are AMPK and AKT signaling pathways. In this context, DPP-4 inhibitors are an emerging class of drugs originally used in the treatment of type II diabetes mellitus and recently proven to show protective effects against various disorders. The protective effect of DPP-4 inhibitors is in part attributed to its antioxidant and anti-inflammatory activities, but most importantly to its ability to modulate AMPK and AKT activities as a result of the inhibition of glucagon-like peptide 1 (GLP-1) breakdown [22, 37].

Accumulating line of evidence shows the negative effects of radiation on the ovaries where it induces ovarian damage with noticeable histopathological deterioration, in addition to a decrease in relative ovarian weight [28, 35, 38]. In our study, control and vilda alone rats showed normal weight gain, and normal histological architecture of the ovary. Rats subjected to γ-radiation showed a marked and significant decrease in both body weight gain and relative ovarian weight. Decreased body weight gain upon radiation exposure was reported in earlier studies [39–41]. This decrease may be attributed to radiation-induced gastrointestinal damage, in addition to the inflammation and cellular stress associated with radiation exposure. In addition, ovarian histopathology was negatively affected as manifested by the atrophied and degenerated ovarian follicles, pyknotic granulosa cell layers, and hyperplasia of the ovarian connective tissue. Co-administration of vilda was able to increase the weight gain rate as compared to irradiated rats and also to reverse some of the histopathological changes as was seen by the multiple follicles at different developmental stages and well-organized graafian follicles. However, it failed to restore the normal relative ovarian weight yet caused a further decrease than the irradiated rats. Of note, vilda-alone treated rats also showed a significant decrease in relative ovarian weight as compared to the control group.

This radiation-induced ovarian damage resulted in a significant decrease in serum estradiol level in irradiated rats as compared to control rats. According to previous studies, the decrease in serum estradiol level is a prominent hallmark of POF owing to the oocytes damage [42, 43]. Vilda co-administration was able to restore the near-normal estradiol levels and showed significant difference from the radiation group. Treatment with vilda alone didn’t show any significant change from the control group.

Although our primary aim was to investigate ovarian failure, exposure of rats to whole body irradiation can adversely affect their brains and impact their behavioral activity [44]. In addition, estrogen is one of the hormones that have neuromodulatory effects [45]. The decreased estrogen level resulting from the ovarian damage would have negative effects on mood, anxiety, and cognition [46]. Hence, it was crucial to explore the changes in behavioral activity in our study. Control group rats showed normal locomotor activity and normal memory acquisition and retrieval ability. Irradiation of rats led to locomotor hyperactivity as was shown by the increased beam interruption count in the locomotor test. Increased locomotor activity after irradiation was previously reported in different studies [47, 48]. In addition, compromised memory was encountered as evidenced by the significantly decreased spontaneous alteration percentage in the Y-maze test and the significantly decreased step-through latency period in the passive avoidance test in the irradiated rats as compared to control rats. Previous studies also reported the negative effects of ionizing radiation on cognition in rats [44, 49]. Vilda co-administration prevented these behavioral changes as was shown by the significantly decreased locomotor activity back to the normal control value and the significantly increased SAP and STL as compared to irradiated rats. This comes in agreement with earlier studies reporting the neuroprotective effects of dpp-4 inhibitors [27, 50].

Besides its effect on cancer cells, radiotherapy can also affect normal cells by inducing DNA damage, which elicits a DNA damage response (DDR) in these cells. This DDR commences with the phosphorylation and activation of ATM, the principal sensor of DNA damage, in particular the DNA double strand breaks (DSBs) [51]. Activation of ATM further stimulates the phosphorylation of AMPK, which is the cellular energy sensor responsible for maintaining metabolic homeostasis [52]. AMPK is expressed in different ovarian cell populations where it regulates primordial follicles’ development and activation [9, 53]. Herein our study, rats exposed to γ-radiation showed significantly elevated pAMPK levels than control rats. This comes in line with previous studies reporting the profound pAMPK elevation after exposure to different stressors as 3-nitropropionic acid, hydrogen peroxide, and ionizing radiation [17, 54, 55]. While this observed pAMPK elevation would usually be beneficial to protect the cells against metabolic stress and help them manage their energy deficits resulting from radiation exposure, this could be the exact reason behind POF in the ovaries as previous studies reported that extensive ovarian AMPK signaling would accelerate follicular atresia [56]. Co-administration of vilda in our study was able to decrease pAMPK levels, however it showed no significant difference from neither control nor radiation groups. Treatment with vilda alone showed no significant difference from the control group.

AMPK activation subsequently results in the activation and overexpression of AKT [15]. Moreover, ionizing radiation was reported to be associated with the upregulation of pAKT levels [10, 57, 58]. At normal levels, AKT is essential for the maintenance of female fertility by regulating follicular growth and differentiation. However, excessive activation and phosphorylation of AKT would accelerate the primordial follicle development process leading to early depletion of the primordial follicle pool, and consequently POF [12, 59]. Irradiation of rats in our study led to a significant elevation of the ovarian pAKT level, leading to a profound acceleration of primordial follicle activation, and this was reflected in the morphometric analysis that showed exhaustion of the primordial follicle pool as compared to the control rats, providing a clue to the radiation-induced POF. In consistence with our findings, a recent study reported a significant elevation of both ovarian AMPK and AKT following exposure to 6 Gy radiation [60]. Interestingly, vilda co-administration also resulted in a significant increase in pAKT levels as compared to control group, together with primordial follicles depletion. Treatment with vilda alone caused a significant increase in pAKT level but much lower than that of radiation + vilda group.

This also may in part explain the observed elevation of serum AMH in our study in radiation and radiation + vilda groups. AMH is a TGF-β family member that is used as a marker to reflect functional ovarian reserve. The functional ovarian reserve refers to the pool of growing and developing follicles which are responsible for AMH production [61]. The profound phosphorylation of AKT upon exposure to radiation and vilda co-administration would activate the entire primordial follicles pool leading to a temporary increase in serum AMH level. In earlier studies, variations in AMH levels were observed in response to hormonal contraception and to vitamin D3 treatment [62–64]. Vilda alone administration caused a slight but insignificantly different increase in serum AMH level as compared to control group.

Both AMPK and AKT act upstream of an eminent transcription factor, Nrf2 [15, 65]. Nrf2 is a redox sensitive basic region leucine zipper transcription factor that acts as a part of the cellular antioxidant defense system. It is usually found in the cytoplasm bound to Kelch-like ECH associated protein 1 (KEAP1), which targets Nrf2 to proteasome-mediated degradation [66]. Increased AMPK and/or AKT activation in turn stimulates the activation of Nrf2. Interestingly, radiation exposure also stimulates the dissociation and upregulation of Nrf2 and its accumulation in the cytoplasm, prior to its translocation to the nucleus. So, cytoplasmic abundance of Nrf2 can be a sign of cellular oxidative stress [67]. Vilda co-administration was able to decrease the cytoplasmic accumulation of Nrf2, reflecting the potential antioxidant effect of vilda. The AMPK/AKT/Nrf2 pathway represents a valuable drug target for protection of multiple organs against different stressors. However, in the ovaries the activation of AMPK/AKT accelerates primordial follicles depletion and follicular atresia, hence leading to radiation-induced POF.

Last but not least, the phosphorylation of AKT also leads to the phosphorylation and activation of CREB. CREB is a nuclear transcription factor that is activated in response to different external stimuli to regulate an array of different physiological processes including cell proliferation, apoptosis, immune response, and neuronal health [68]. Exposure to ionizing radiation stimulates the phosphorylation of both AKT and CREB to promote cell survival [57]. In our experiment, irradiated rats had significantly higher pCREB levels than control rats. This comes in accordance with previous studies highlighting the role of pCREB in primordial follicle activation [69]. While normally, increased cell survival would be expected upon AKT and CREB activation, their activation in the ovaries led to global activation of primordial follicles pool. Those primordial follicles once activated cannot go back to the dormant state, leading them to their final fate -atresia.

## Conclusion

To review, POF generally results from either direct follicular loss or an accelerated follicular activation rate, ultimately leading to follicular depletion. Both AMPK and AKT play key roles in ovarian follicles’ activation and development; however, to date, no study examined their mutual association with POF pathogenesis. Our findings indicate that ovarian exposure to γ-radiation led to a faster-than-normal rate of primordial follicles’ activation through the activation and upregulation of pAMPK, which consequently led to the activation and phosphorylation of AKT, a kinase that would normally promote follicular development, but in the presence of radiation, it markedly increases the follicular activation rate. To the best of our knowledge, our study was the first to highlight the role of AMPK/AKT pathway in the pathogenesis of radiation-induced POF by causing a premature depletion of the primordial follicles pool. Vilda co-administration in irradiated rats somehow preserved the ovarian histopathological architecture but failed to maintain the primordial follicle pool. While this issue might require further investigations and studies, women undergoing radiotherapy should be advised to avoid the use of drugs that activate AKT, of which vildagliptin was the one we investigated, during their treatment course.

## Data Availability

No datasets were generated or analysed during the current study.

## Abbreviations

AMH: Anti-müllerian hormone
E2: Estradiol
GSI: Gonadosomatic index
pAMPK: Phosphorylated Adenosine monophosphate-activated protein kinase
pAKT: Phosphorylated AKT
pCREB: Phosphorylated cAMP Response Element-Binding Protein
POF: Premature ovarian failure
Vilda: Vildagliptin
